# SPECT- and PET-Based Approaches for Noninvasive Diagnosis of Acute Renal Allograft Rejection

**DOI:** 10.1155/2014/874785

**Published:** 2014-04-01

**Authors:** Helga Pawelski, Uta Schnöckel, Dominik Kentrup, Alexander Grabner, Michael Schäfers, Stefan Reuter

**Affiliations:** ^1^Department of Medicine D, Experimental Nephrology, University of Münster, Albert-Schweizer Campus 1, 48149 Münster, Germany; ^2^Department of Nuclear Medicine, University of Münster, Albert-Schweizer Campus 1, 48149 Münster, Germany; ^3^European Institute for Molecular Imaging, University of Münster, Albert-Schweizer Campus 1, 48149 Münster, Germany

## Abstract

Molecular imaging techniques such as single photon emission computed tomography (SPECT) or positron emission tomography are promising tools for noninvasive diagnosis of acute allograft rejection (AR). Given the importance of renal transplantation and the limitation of available donors, detailed analysis of factors that affect transplant survival is important. Episodes of acute allograft rejection are a negative prognostic factor for long-term graft survival. Invasive core needle biopsies are still the “goldstandard” in rejection diagnostics. Nevertheless, they are cumbersome to the patient and carry the risk of significant graft injury. Notably, they cannot be performed on patients taking anticoagulant drugs. Therefore, a noninvasive tool assessing the whole organ for specific and fast detection of acute allograft rejection is desirable. We herein review SPECT- and PET-based approaches for noninvasive molecular imaging-based diagnostics of acute transplant rejection.

## 1. Introduction

Noninvasive imaging techniques for medical diagnosis allowing visualization of specific biological processes have made tremendous progress in the last decades. One major issue focused on the advances in sensitivity and specificity but also on compatibility, accessibility, and affordability.

Scintigraphy, single-photon emission computed tomography (SPECT), and positron emission tomography (PET) are imaging procedures based on the detection of internal radiation (mostly intravenous injection of a radioactive tracer), where gamma rays emitted by tracers containing radionuclides (directly in gamma scintigraphy and SPECT or indirectly (annihilation) in PET) are captured via an external detector system (gamma camera). Nuclear imaging has the benefits of high intrinsic sensitivity, recognising targets present in a very low concentration, exceptional penetration of tissues, whole-body visualisation, and a large range of available, clinically tested tracers and consequently a high specificity [1, 2].

While planar scintigraphy produces two-dimensional pictures, SPECT and PET offer the possibility to generate three-dimensional images allowing higher resolution and therefore better monitoring of the deposition and clearance of the employed tracers. The difference of SPECT and PET lies in the detection procedure, resolution, and utilized radionuclides. The spatial resolution is restricted to 3–5 mm in PET and 8–10 mm in SPECT; however, detection of the contrast agent is even possible in the nano- and picomolar range [3]. Although PET offers better quantification and a 2- to 3-fold superior sensitivity than SPECT, the latter still reflects the most commonly used imaging technology due to cost-effectiveness, availability, and existence of a broader array of adequate radionuclides [4]. Both imaging techniques generate functional images of metabolic processes and not morphological visualisation. Therefore, these modalities are utilized for evaluation of the function of the examined organ and illustration of molecular and cellular events like apoptosis, inflammation, infection, change in pH, and metabolism [5]. This review focuses on the principles of SPECT and PET and their field of preclinical and clinical application and gives an overview of their potential mode of operation diagnosis of acute (renal) allograft rejection (AR).

## 2. Radiopharmaceuticals

In order to receive expedient information about the biological process of interest, the applied radiotracers have to fulfil a variation of features. First, the utilized contrast agent needs to bind sufficiently to the target of interest, delivering a clear signal greater than the background noise of the surrounding tissue (good signal-to-noise ratio) [5]. Appropriate tracers have a great binding specificity for molecular targets, upregulated under specific pathologic settings, permitting comparison to the physiological state. Further, a good tracer allows visualisation in very low concentration and of low concentrated targets, limiting its side effects. The more specific the marker for a condition or a disease is, the more precise the information obtained is. Fast clearance of the used radionuclides is also an aspect that needs to be taken into consideration. Otherwise, distinction between specific and unspecific signal is not possible [5]. Distinct radiopharmaceuticals have been developed for SPECT and PET, nicely overviewed by Signore and Fani [2, 6].

In PET, the predominant radionuclide used for tracers depicts ^18^F, but other markers to visualize biological processes like ^11^C, ^13^N, ^15^O, ^68^Ga, ^64^Cu, ^60^Cu, ^86^Y, ^89^Zr, and ^124^I have been developed. ^18^F-based tracers utilized in PET directly incorporate the radionuclide into the biomolecule via substitution making it almost undistinguishable from their nonradioactive analogs by displaying the same characteristics [7]. Depending on the target of interest (e.g., proteins and cellular processes), a suitable compound (peptide, proteins, antibodies, or small molecules), serving as basis for the tracer, needs to be selected [8]. Not only should the duration of the process of interest correspond to the half-life of the chosen radionuclide but also the emitted energy of the positrons needs to be considered for the spatial resolution (low energy and better spatial resolution) [8]. The radiopharmaceutical most commonly used in clinics is ^18^F-FDG (with a comparable metabolism to normal glucose but trapping of ^18^F-FDG in the cell) for determination of metabolic activity, inflammatory sites, and oncologic analysis.

^99m^Tc, ^111^In, ^67^Ga, and ^123^I depict the deployed markers for SPECT imaging. Among these ^99m^Tc represents the radionuclide with the largest area of application due to its optimal decay characteristics, easy production, and availability [7]. Compared to PET tracers, the generally longer half-lives of these SPECT radionuclides constitute an advantage correlating better with the half-lives of biological processes. In addition, these radiotracers are generally available. Furthermore, ^99m^Tc-based tracers are easy to produce by a generator and do not need a nearby medical cyclotron [9] in contrast to rather short-lived unstable PET tracers. However, SPECT tracers also show some disadvantages. Incorporation of ^99m^Tc, for example, into a molecule is more complex than the substitution of ^18^F in PET, involving chelating moieties and leading to possible steric hindrance [7]. Therefore, ^99m^Tc-based labelling is not suitable for imaging every process. It has been employed for visualisation of bone and joint infections, while ^67^Ga was successfully applied in tumor imaging [10]. For identification of hypoxic areas different tracers usable for standard gamma cameras have been developed such as ^123^I-labelled iodoazomycin arabinoside and different ^99m^Tc-based compounds [11–13].

## 3. Labelling of Intracellular Targets 

Gallagher et al. reported already in 1978 the potential of ^18^F-FDG for scintigraphic detection of glucose metabolism [14]. The cellular uptake of the radiolabelled glucose analog, which has a similar metabolic route as glucose, via glucose transporters (e.g., GLUT1) correlates with the metabolic activity of the cell and therefore with the cells energy demand. In the cell, the hexokinase phosphorylates ^18^F-FDG into ^18^F-FDG-6-phosphate. Because of the inability of the glucose-fructose isomerase to transduce phosphorylated ^18^F-FDG, it is metabolically trapped in cells displaying high metabolism and allowing visualization of ^18^F-FDG-biodistribution [15]. While the technique remained constant, the detection procedure was refined with PET. By now, assessment of metabolic activity via evaluation of glucose metabolism depicts a clinically well-established method.

It has to be noted that ^18^F-FDG uptake is not a specific event, meaning that no specificity for a disease or target exists. Its uptake is related to tissue metabolism and presence of glucose transporters. Since stimulated inflammatory cells as well as tumor cells are metabolically very active and show an increased expression of glucose transporters, ^18^F-FDG can be deployed to reveal sites of inflammation and tumors.

Furthermore, the glucose transporters exhibit an elevated affinity for deoxyglucose in an ongoing inflammatory state. In fact, ^18^F-FDG has been successfully applied for clinical and preclinical diagnosis in many pathologic settings like cancer [15–18], inflammatory diseases such as atherosclerosis [19–21], arterial inflammation [22], psoriasis [23], transplantation medicine [24–28], asthma [29, 30], and fibrosis [31].

Kidney transplantation represents the therapy of choice for patients suffering from end-stage renal failure. Despite exceeding advances in transplantation medicine throughout the last decades, allograft rejection still depicts a central issue of graft failure. The leading cause of death-censored graft loss is either humoral-mediated or T cell-mediated allograft rejection. In this context, acute rejection (AR) episodes have a great impact on the survival of the transplant. Since AR correlates with the constraint of graft function, it is of major importance to counteract its effect immediately. Therefore, early detection of AR is essential. Different techniques to address this aspect have been developed and until today core-needle biopsy still constitutes the gold standard among these. However, considering the invasiveness of this method it would be beneficial if noninvasive imaging methods were clinically available which do not pose a risk for the grafts integrity. We have demonstrated an interesting noninvasive approach monitoring the uptake of ^18^F-FDG [27]. In PET, visualisation of the transplant's function as well as cellular and molecular processes characteristic for AR like leukocyte recruitment, restriction of renal activity, hypoxia, and cell death is possible. Because of high metabolism due to infiltration of inflammatory cells, a specific ^18^F-FDG-uptake pattern arises, allowing determination of AR, while at the same time discriminating fundamental differential diagnosis in a rat renal allograft model [27].

**Figure 1:**
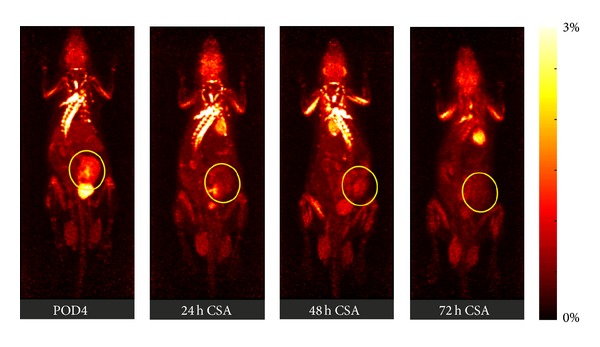
A series of PET images of dynamic whole-body acquisitions 180 min after a single tail vein injection of ^18^F-FDG into an allogeneically kidney-transplanted rat on postoperative day 4 (POD4). After development of acute rejection (the allograft shows an intense ^18^F-FDG uptake on POD4) the recipient was treated with cyclosporine A showing already 24 h after commencement of immunosuppressive therapy a significant decrease of the ^18^F-FDG uptake into the renal parenchyma (≜ therapy response). Please note that urine in the renal pelvis can contain eliminated ^18^F-FDG. Therefore, it should be excluded from the assessments. Renal graft is marked with yellow circle. % ID: % of injected dose. The figure was adapted from Reuter et al. [28].

In transplantation medicine, imaging of ^18^F-FDG uptake has great potential to become a new method to accurately determine and monitor rejection episodes (Figure 1) [27]. One fact concerning the clearance of ^18^F-FDG needs to be taken into consideration. Elimination of ^18^F-FDG happens via the urine causing drainage of the radiolabelled glucose into the pelvis. This leads to a false positive signal which can be significantly reduced by extending the time between tracer application and tracer detection (late acquisition), ensuring elimination of excessive tracer [27]. For clinical use, further studies and careful assessment of risks for the patient need to be performed.

As mentioned before, ^18^F-FDG uptake is a nonspecific process. ^18^F-FDG is administered intravenously to monitor biodistribution subsequently. A great amount of ^18^F-FDG is needed in order to receive a clear signal of enhanced metabolic activity since all cells exhibit metabolism to some extent.

Because of the clinical importance of ^18^F-FDG as a diagnostic tool, the development of inexpensive and readily available glucose analogs applicable in SPECT was supported. ^99m^Tc-based glucose labelling was performed resulting in the formation of glucose tracers failing to exhibit similar characteristics as ^18^F-FDG. Transport of these glucose analogs into the cell was not mediated through GLUT1 nor did phosphorylation take place via hexokinase. Since these features are essential for accumulation of the glucose analogs in metabolic active cells, the ^99m^Tc-labelled glucose is not feasible for imaging of metabolism and fails to substitute ^18^F-FDG. A reason for this could be a sterical hindrance of the GLUT1 binding site through the ^99m^Tc tag [4].

## 4. *Ex Vivo* Labelling of Leukocytes

Inflammation is a process occurring in many distinctive disease settings such as autoimmune diseases, infections, and allograft rejection. During an ongoing inflammation, specific cells including lymphocytes, granulocytes, and macrophages are activated and recruited to the inflammatory site where they infiltrate the affected tissue. Approaches to visualise these infiltrating cells and therefore to image inflammation have been performed via radiolabelled white blood cells (WBC) and it has been shown that autologous leukocytes radiolabelled with either ^99m^Tc-HMPAO or ^111^In-oxine for SPECT or ^18^F-FDG or ^64^Cu for PET, respectively, specifically are enriched in inflamed tissues [32–34]. In fact, this is an established clinical procedure used in many different pathologic settings such as Crohn's disease [35], osteomyelitis [36], fever of unknown origin, infection [37], acute appendicitis [38], and arterial and colonic inflammation [39].

For successful imaging of radiolabelled cells a few aspects need to be taken into account. First, regarding the applied cells, it is notable that, before entering the reticuloendothelial system consisting of spleen, liver, and bone marrow and centre of acute and chronic inflammation through the blood pool, the administered labelled WBC accumulate briefly in the lung. Second, while the biodistribution of the radioactivity in normal human subjects forms a similar pattern independent of the radionuclide (^18^F-FDG, ^111^In, and ^99m^Tc) used [40], the labelling stability varies. This aspect is important to ensure that the captured radioactive signal reflects WBC accumulation and that signal artefacts due to free (i.e., not cell bound) tracers are eliminated. Third, compound stability of the tracer and radionuclide half-life have to be taken into account. ^18^F-labelling exhibits the lowest compound stability and a radioactive half-life of 109 min. Due to this short half-life, long-time stability of this tracer is not of clinical interest. For processes with a longer duration, other radionuclides should be considered for labelling (^99m^Tc-HMPAO half-life = 66 h).

Furthermore, labelling efficiency and viability of marked cells reveal discrepancies when distinct radionuclides were deployed. Labelling with ^111^In-oxine and ^64^Cu leads to an approximate efficiency of 80% while the labelling rate of ^18^F-FDG yields in 60% [41]. However, since labelling of cytotoxic T cells reaches different efficiencies (^111^In-oxine (68%), ^18^F-FDG (64%), and ^99m^Tc-HMPAO (31%)), labelling potential seems to be cell-type dependant [42]. Another important issue is viability of marked cells. Studies have addressed this factor and demonstrated that similar viability rates are reached with ^111^In-oxine-,^99m^Tc-HMPAO-, ^18^F-FDG-, and ^64^Cu-labelling approaches in the first four hours [41]. However, after a time period of 24 hours, a sharp decline of cell survival was observed restricting long-time monitoring of specific processes with a single-dose application.

**Figure 2:**
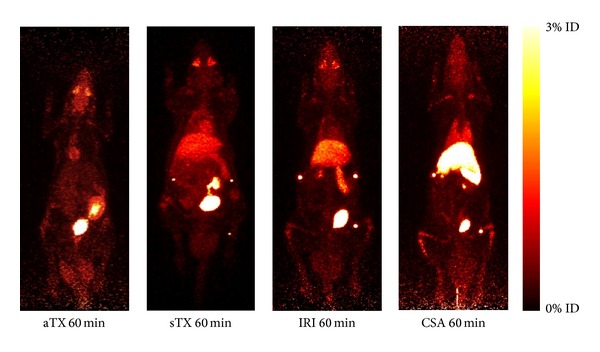
Exemplary PET images (day 4 after surgery) of dynamic whole-body acquisitions of allogeneically (aTX) and syngeneically transplanted (sTX) rats, rats with ATN (IRI), and rats with acute cyclosporine toxicity (CSA). Effects are summarized after tail vein injection of ^18^F-FDG—labeled T cells (maximum-intensity projection, whole-body acquisition for 20 min at 60 min (50–70 min after injection). On postoperative day 4 aTX kidneys exhibited significantly elevated ^18^F-FDG uptake in comparison to native controls. Accumulation of labelled cells in kidneys with IRI or acute CSA toxicity and sTX was not significantly different from native controls. Please note that the renal pelvis can contain eliminated ^18^F-FDG/^18^F-fluoride. Therefore, it has to be excluded from the measurements. ID: injected dose. The figure was taken from Grabner et al. [44].

In preclinical and clinical studies labelled leukocytes were utilized for detection of acute rejection in different organs (intestine, heart, skin, or kidney). In kidney-transplanted patients, scintigraphic analysis of ^99m^Tc-labelled mononuclear cells allowed diagnosis of AR and discrimination from acute tubular necrosis (ATN) [43]. In a more sophisticated approach with PET, human leukocytes, in particular human cytotoxic lymphocytes, marked with low amounts of ^18^F-FDG* ex vivo* were applied in a rat kidney-transplant model [44]. Subsequent PET analysis led to the differential diagnosis of AR excluding other causes of early graft dysfunction such as ischemia, ATN, or immunosuppressive toxicity (Figure 2).

## 5. *In Vivo* Labelling with Antibodies

Visualisation of inflammation without* ex vivo* labelled leukocytes is possible via another approach utilizing radiolabelled monoclonal antibodies (mAb) directed against infiltrating cells. On first sight, many advantages are connected to this procedure. On the one hand, a vast quantity of targeting possibilities exists, allowing, at least theoretically, high specificity and a reasonable signal-to-noise ratio. On the other hand, the procedure of antibody production is standardized coming along with easy storage, simple administration, and cost-effectiveness [2]. In spite of many advantages of this method, some problems concerning target accessibility and safety exist. Since the antibodies are not able to penetrate tissues, targeting is restricted to intra- and perivascular antigens, limiting their range of action. Furthermore, allergic complications can arise when administering the antibodies to a patient. Additionally, since antibodies are able to elicit an immune response, their immunogenicity needs to be determined before their usage in order to prevent falsification through their activity.

Numerous radiolabelled mAb and mAb fragments have been designed to address infiltrating cells. Since T cells and B cells constitute cell types playing a major role during inflammation, antibodies directed against CD3, CD4, CD8, CD25, CD20, and granulocytes have been developed. They are mainly marked with ^99m^Tc, ^111^In, and ^123^I [2].

In transplantation medicine a few approaches have been performed to elucidate rejection episodes. Antibodies directed against the CD3 complex, expressed on T cells, natural killer cells (NK), and natural killer T cells (NK T), have been designed to illustrate T-cell infiltration and thus to determine AR. In a preliminary study, Martins et al. applied ^99m^Tc-OKT3 to successfully diagnose AR in kidney-transplanted patients [45]; however, more data is needed to confirm this finding. A clear disadvantage of this antibody is a side effect due to immunogenicity. A more biocompatible humanized alternative but still in need of further evaluation is the radiolabelled CD3 antibody ^99m^Tc-SHNH-visilizumab.

A vast number of possibilities exist to image targets of interest accessible via the vascular system. In order to deploy them for diagnosis of different diseases, identification of specific markers upregulated during distinct pathologic processes is necessary. Antibodies directed against vascular proteins such as vascular adhesion molecule-1 (VCAM-1), intercellular adhesion molecule-1 (ICAM-1), or selectins could be used to address vascular-related diseases such as atherosclerosis and visualise the sites of thrombus formation.

The greatest problem that needs to be overcome in this setting is the clearance of unbound radiolabelled antibody in order to improve the background-to-noise ratio. Since free tracer circulates in the vascular system, producing a SPECT or PET signal, the signal arising from the bound antibody might be masked depending on the ratio of free-to-bound antibody. Moreover, the high molecular weight of most mAb constitutes a limitation since sequestration by specific cells might result in diminution of available, free mAb able to bind to the target of interest. On the contrary, mAb tracers with low molecular weight might produce unspecific signals due to their passive diffusion into and enrichment in inflamed tissues. A possible solution of this problem could be the selection of radiolabelled antibody fragments with intermediate molecular weight (40–80 kDa). These have been demonstrated to exhibit features ideal for PET and SPECT tracers such as low immunogenicity, fast blood clearance, better enrichment in sites of inflammation, and no unspecific binding to Fc receptors due to the absence of the Fc part [2].

## 6. Conclusion

Specific noninvasive methods for diagnosis of AR in transplant patients are highly in demand. Molecular and cellular imaging strategies using SPECT- or PET-based approaches have great potential to fulfil these requests of allograft surveillance. Advances in technology and tracer development open new possibilities in regard to diagnosis and management of renal rejection. Currently, nearly all of these promising new approaches are still at an experimental stage. Future studies will elucidate whether these diagnostics tools are transmissible to transplant patients in clinical routine.
